# The association between dietary knowledge based on the *Chinese Dietary Guidelines* and adherence to healthy dietary habits: a large-scale cross-sectional study

**DOI:** 10.3389/fpsyg.2024.1453815

**Published:** 2024-10-18

**Authors:** Zhongyu Ren, Zixuan Hao, Jianhua Cao

**Affiliations:** ^1^School of Physical Education, Southwest University, Chongqing, China; ^2^Department of Physical Education, Chongqing Institute of Foreign Studies, Chongqing, China

**Keywords:** dietary knowledge, *Chinese Dietary Guidelines*, dietary habits, cross-sectional, Chinese

## Abstract

**Introduction:**

Previous systematic review has shown that individuals with more comprehensive dietary knowledge tend to engage in healthier eating patterns among American or European population. However, research on the association between dietary knowledge based on the *Chinese Dietary Guidelines* and healthy dietary behaviors, particularly among adolescents and college students in China, is lacking. This study aimed to examine the association between dietary knowledge based on the *Chinese Dietary Guidelines* and adherence to healthy dietary behaviors among adolescents and college students in China.

**Methods:**

A cross-sectional study was conducted in China in August and October 2023. The study involved 527 adolescents and 11,856 college students. A convenience and cluster sampling methodology was employed to select one or two grades from 33 different university majors. The dietary behaviors of college students were evaluated by assessing their consumption of nine food groups: water, eggs, milk and milk products, vegetables, fruit, red meat, soy and soy products, seafood, and sugar-sweetened beverages. The dietary behaviors of adolescents were evaluated by assessing their consumption of five food groups: fast food, salty snack foods, fruits, vegetables, and soft drinks and sugared fruit beverages. The participants’ dietary knowledge was assessed using the *Chinese Dietary Guidelines*. The relationship between dietary knowledge and behaviors was examined using a multivariate logistic regression analysis.

**Results:**

The questionnaire response rate was 100.0%. Multivariate logistic regression analysis revealed a significant positive association between dietary knowledge and the likelihood of exhibiting diverse dietary behaviors among college students. After adjusting for gender, age, family income, place of residence, and parents’ education levels, the results demonstrated a positive association between dietary knowledge and adherence to 4–8 eating habits among college students. In contrast, similar association was not observed among adolescent.

**Conclusion:**

This study revealed a significant association between dietary knowledge based on the Guidelines and adherence to healthy dietary behaviors among college students in China. That is to say, the higher the level of dietary knowledge based on the Guidelines among college students, the healthier the dietary behaviors they tend to adopt in their daily lives. These findings indicate the necessity of developing educational interventions based on the Guidelines to enhance dietary knowledge among individuals with limited dietary knowledge. Such interventions could facilitate the acquisition of essential health-related knowledge and strengthen motivation to engage in healthy dietary behaviors. Future studies should employ longitudinal prospective designs or randomized controlled trials in order to establish a causal association between dietary knowledge based on the Guidelines and healthy dietary behaviors.

## 1 Introduction

With the sustained development of the economy and steady improvement of living standards, significant changes have occurred in the dietary habits of Chinese residents. High-calorie and high-fat diets have become increasingly popular. This shift has raised public health concerns. Studies have shown that nearly one-fifth of the global population is at risk of chronic non-communicable diseases, such as hypertension and diabetes, due to poor dietary habits (Afshin et al., 2019). Conversely, a balanced diet is associated with lower mortality (Shan et al., 2023) and increased lifespan (Fadnes et al., 2023), highlighting the urgent need to promote healthy dietary patterns in medicine and nutrition science.

College is the starting point for independent daily life(Vila-Martí et al., 2021; Ma, 2011) and defining moment for acquiring dietary autonomy and shaping dietary behaviors that could be sustained into adulthood(Winpenny et al., 2018). A longitudinal study found that the intake of vegetables and fruits decreased between the ages of 14 and 23 years and peaked around the age of 30 years, whereas the intake of sugary drinks and candy peaked around the age of 18 years and then began to decline (Winpenny et al., 2018). Another longitudinal survey of individuals aged 30–59 years revealed that healthy dietary patterns increased with age (Talegawkar et al., 2020). Unhealthy eating habits in early life had adverse effects on long-term health outcomes, including Type 2 diabetes (Malik et al., 2012), hypertension (Qiufen et al., 2022), colorectal cancer (Thordardottir et al., 2022), and arterial stiffness (Van de Laar et al., 2013). This suggests that exploring the dietary behaviors of college students and identifying the influencing factors is significant for understanding and improving their nutritional health.

The *Chinese Dietary Guidelines* (hereafter, the *Guidelines*) present a scientific consensus on healthy dietary behaviors and have informed nutrition promotion and health initiatives of health education workers, policymakers, and other stakeholders. The *Guidelines* are intended to help individuals make informed food choices and promote physical activity to maintain health and prevent nutrition-related diseases (Wang, 2021). Since the last century, nutrition education programs have been disseminated globally through schools (Al-Jawaldeh et al., 2023), government agencies (Ukam and Otareh, 2019), and health promotion organizations (Robles et al., 2019), aiming to improve residents’ understanding of nutrition and encourage balanced diets. However, research has not explored the general population’s nutritional knowledge, particularly regarding government-developed dietary guidelines and core food group recommendations, and its impact on dietary behavior. Thus, an understanding of how nutritional education translates into healthy dietary behaviors in daily life is lacking.

Previous systematic review has shown a positive association between nutritional knowledge and dietary behaviors among American or European population (Spronk et al., 2014). However, not all studies observed this relationship. Moreover, although studies have shown a positive association between knowledge of dietary guidelines and healthy dietary habits, these studies focused on single dietary behaviors among primary and middle school students (Feng Peng et al., 2019) and the general adult population(Huang FeiFei et al., 2015). The Chinese government and health experts have long worked to develop effective measures to improve dietary behavior. For example, the Government of China advocates the implementation of nutritional health science and education campaigns, including National Nutrition Week, National Food Safety Awareness Week, National Student Nutrition Day and National Iodine Deficiency Disease Prevention and Control Day (GOOTS Council, 2017). However, due to the imbalance between theoretical research and practical implementation, new interventions to promote healthy dietary behavior have been continuously called for. The National Nutrition Plan (2017–2030) requires disseminating dietary guidelines and nutritional health knowledge to various groups (Yan, 2017).

Therefore, this study examined the relationship between knowledge of dietary guidelines and dietary behaviors considering multiple food groups among secondary school and college students in China. A comprehensive understanding of this relationship is essential for developing nutrition education strategies and public health policies.

## 2 Methods

### 2.1 Research design and population

This study adopted a cross-sectional research design. A questionnaire survey was conducted at the physical fitness testing centers of four college (two in Sichuan Province and two in Chongqing Municipality) and 11 college (six in Liaoning Province, one in Jilin Municipality, and four in Chongqing Municipality) in China in August 2023 and October 2023, respectively (Ma et al., 2024). The present investigation was carried out using a stratified random sampling method, which accounted for sampling error and the stratification factor (stratified by grade). A random sample of 1–2 grades was selected from 33 majors drawn from 11 universities in accordance with the grade stratification and random number table method. A total of 11,856 university students, with a median age of 19.0 years, were sampled, and the response rate was 100.00%. Written informed consent was obtained from all participants. For participants aged below 16, parental consent was obtained following a comprehensive evaluation. This study was approved by the Ethics Committee of the School of Physical Education of Southwest University.

The China Health and Nutrition Survey (CHNS), which was established in 1989, is an ongoing prospective cohort study. A previous study has published a detailed study design (Du et al., 2013). In brief, the multi-stage random cluster sampling method was used to select 4,400 households and 19,000 participants, covering nine provinces (Guizhou, Guangxi, Heilongjiang, Henan, Hubei, Hunan, Liaoning, Jiangsu, and Shandong). This study utilized the 2015 CHNS data and included individuals aged 18 years and younger as study subjects (n = 2,742). After removing individuals with missing sociodemographic characteristics, and diet-related variables, a total of 527 subjects were involved in the final analysis. Publicly available datasets were analyzed in this study. This data can be found here: https://www.cpc.unc.edu/projects/china (CCFDCA Prevention, 2015). Prior to the commencement of the study, ethical approval was obtained from the Institutional Review Board of the University of North Carolina at Chapel Hill and the National Institute for Nutrition and Health, Chinese Center for Disease Control and Prevention.

### 2.2 Assessment of eating behaviors

Food preferences are an indicator of one’s attitude and consumption patterns with regard to specific food types (Ma et al., 2021), and an individual’s food preferences have the potential to significantly influence their long-term health outcomes (Wang et al., 2024). Food preferences were assessed by five questions in the CHNS questionnaires (Wang et al., 2024). Adolescents were asked to rate their preference for five types of food items: fast food (e.g., pizza, burgers), salty snacks (e.g., potato chips), fruits, vegetables, and soft drinks and sugared fruit beverages. Responses were rated on a five-point Likert scale (1 = strongly dislike, 5 = strongly like). These scores were converted into binary variables: scores of 4 and 5 were classified as liking the food, whereas scores of 1–3 were classified as reflecting dislike or neutrality (Ma et al., 2021).

The dietary behaviors of college students were assessed using the adult version of the Chinese Dietary Pagoda (CN Society, 2022). Participants were asked to report their dietary habits regarding the intake of water, eggs, milk and milk products, vegetables, fruits, red meat, soy and soy products, and seafood. An additional item on sugar-sweetened beverages was derived from a prospective cohort study that showed a 21% higher risk of premature death among individuals who consumed ≥2 cups of sugar-sweetened beverages per day compared to those who consumed <1 cup per month (Gitanjali, 2015). Water consumption was assessed using one question: “How much water do you consume on average per day?” Responses were rated on a six-point scale (< 250 mL, 250–500 mL, 500–1,000 mL, 1,000–1,500 mL, 1,500–2000 mL, and > 2000 mL) and categorized as regular (≥ 1,500 mL) or irregular (< 1,500 mL) water intake. Sugar-sweetened beverage intake was assessed using one question: “On average, how many sugar-sweetened beverages do you drink per day?” Responses were rated on a five-point scale (none/never consume, < 500 mL, 500–1,000 mL, 1,000–1,500 mL, and > 1,500 mL) and categorized as ingesting or not ingesting. Egg intake was assessed using one question: “How many eggs do you eat on average per day?” Responses were rated on a five-point scale (0/never consume, 1, 2, 3, 4, and > 4) and categorized as regular (≥ 1) or irregular (0) consumption. Milk and dairy product intake was assessed using one question: “How much milk and dairy products do you consume on average per day?” Responses were rated on a five-point scale (none/never consume, < 250 mL, 250–500 mL, 500–750 mL, and > 750 mL) and categorized as regular (≥ 250 mL) or irregular (< 250 mL) consumption. Vegetable and fruit intake was assessed using one question: “On average, how many times a day do you eat vegetables or fruit?” Responses were rated on a five-point scale (never, once, twice, three times, four times, and more than four times) and categorized as regular (once or more) or irregular (never) consumption. Red meat, soy, and fish intake was assessed using one question: “On average, how many times a week do you eat red meat (including pork, beef, lamb, and processed products such as bacon and sausages), soy products, or fish products?” Responses were rated on an eight-point scale (less than once/never consume, once, twice, three times, four times, five times, six times, and daily) and categorized as regular (twice or more) or irregular (less than twice) consumption.

For each food type, responses categorized as regular consumption were assigned a score of 1, whereas those categorized as irregular were assigned a score of 0. Total scores were obtained by summing scores for all food types and ranged from 0 to 9, with higher scores indicating healthier eating habits. The reliability of the participants’ reported dietary behaviors over time was quantified using the intra-group correlation coefficient (ICC). The ICC values were statistically significant and ranged from 0.947 to 0.987, indicating high retest reliability of the assessed dietary behaviors.

### 2.3 Assessment of dietary knowledge based on *Guidelines*

The participants’ dietary knowledge was assessed using the Dietary Knowledge of Chinese Residents scale (Yang et al., 2020). Responses were rated on a five-point Likert scale (1 = strongly disagree, 5 = strongly agree). These scores were converted into binary variables: scores of 4 and 5 were classified as good dietary knowledge and coded as 1, whereas scores of 1–3 were classified as poor dietary knowledge and coded as 0. Total scores were obtained by summing the individual item scores, with higher total scores indicating greater dietary knowledge. The study population were divided into quartiles based on dietary knowledge scores to compare different levels of knowledge. The Cronbach’s alpha coefficients were 0.749 and 0.842 for adolescents and college students, respectively, indicating robust internal consistency reliability.

### 2.4 Statistical methods

All data were imported into an Excel database for organization and generalization. SPSS 23.0 was used for statistical analysis. All categorical data were expressed as percentage (%) and continuous variables were presented as medians [interquartile range (IQR)] due to the non-normal distribution of the data. The chi-square test for categorical variables and the Kruskal-Wallis test for continuous variables were employed to assess the differences in participant characteristics between individuals with categories of dietary knowledge.

We used multivariate logistic regression to examine the association between dietary knowledge and healthy eating behaviors. Dietary knowledge, categorized into quartiles (Q1–Q4), was the independent variable, whereas healthy eating behaviors were the dependent variable. To compare the differences in the likelihood of healthy eating behaviors between groups with different levels of dietary knowledge, odds ratios (ORs) and 95% confidence intervals (CIs) were calculated for Q2, Q3, and Q4, using the Q1 level as the reference group. To examine the effects of potential confounders, two statistical models were constructed for the adolescent: Model 1 was a crude model, and Model 2 was adjusted for gender, age, annual family income, residence, education level. Similarly, two statistical models were constructed for the college student population: Model 1 was a crude model, and Model 2 was adjusted for gender, age, annual household income, place of residence, father’s education level, and mother’s education level. Moreover, we separately analyzed the association between dietary knowledge and the likelihood of consuming each food type in both Models 1 and 2. All statistical analyses were performed using two-sided tests, with *p* < 0.05 indicating statistical significance.

## 3 Results

### 3.1 Participants’ characteristics

Table 1 presents the characteristics of the college students. This study included 11,856 college students (33.2% men, 66.8% women, median age = 19.0 years). Among them, 58.9% resided in rural areas, with over one-third (34.6%) reporting an annual household income of over 35,000 yuan. Approximately one-third of the students had parents with a bachelor’s degree or higher. Moreover, individuals with higher levels of dietary knowledge tended to be men (*p* < 0.001), be older (*p* < 0.001), and have parents with higher educational levels (*p* ≤ 0.007).

**Table 1:** Participants’ characteristics according to categories of dietary knowledge based on the Guidelines score (college students).

*N* = 11,856	Q1	Q2	Q3	Q4	*p* value
Gender, male	29.1	27.4	34.6	42.7	<0.001
Age, year	19.0 (18.0, 19.0)	18.0 (18.0, 19.0)	19.0 (18.0, 19.0)	19.0 (18.0, 19.0)	<0.001
Annual family income, %					
<20,000 yuan	36.6	35.7	34.8	34.9	0.137
20,000–35,000 yuan	27.9	30.8	30.6	30.5	
>35,000 yuan	35.5	33.5	34.7	34.6	
Residence, %					
Town	41.8	40.2	41.7	40.8	0.516
Rural	58.2	59.8	58.3	59.2	
Father’s education level, %					
Primary and below	22.8	24.4	23.0	25.5	0.007
Junior high	38.4	40.4	42.0	37.5	
High school	22.6	20.7	20.6	21.6	
Bachelor degree and above	16.1	14.5	14.4	15.3	
Mother’s education level, %					
Primary and below	29.7	32.8	32.2	33.2	0.002
Junior high	36.8	35.8	38.5	34.4	
High school	19.5	19.1	17.1	18.5	
Bachelor degree and above	14.0	12.3	12.1	13.8	

Table 2 presents the characteristics of the adolescents. This study included 527 adolescents (51.6% men, 48.4% women, median age = 14.0 years). Among them, 57.3% resided in rural areas, and 63.9% reported an annual household income of over 35,000 yuan. Approximately 1.5% had parents with a bachelor’s degree or higher. Moreover, individuals with higher levels of dietary knowledge tended to have higher income levels (*p* = 0.043).

**Table 2:** Participants’ characteristics according to categories of dietary knowledge based on the Guidelines score (adolescent).

*N* = 527	Q1 (*n* = 143)	Q2 (*n* = 136)	Q3 (*n* = 176)	Q4 (*n* = 72)	*p*- value
Gender, male	53.1	58.1	46.0	50.0	0.196
Age, year	14.0 (13.0, 15.0)	14.0 (12.0, 16.0)	14.0 (13.0, 16.0)	14.0 (12.0, 16.0)	0.056
Annual family income, %					
<20,000 yuan	22.4	19.1	15.9	27.8	0.043
20,000–35,000 yuan	16.1	22.8	13.1	9.7	
>35,000 yuan	61.5	58.1	71.0	62.5	
Residence, %					
Town	45.5	44.1	41.5	37.5	0.692
Rural	54.5	55.9	58.5	62.5	
Education level, %					
Primary and below	46.2	38.2	37.5	36.1	0.318
Junior high	35.7	37.5	39.8	44.4	
High school	9.1	10.3	14.2	11.1	
Bachelor degree and above	0.0	3.7	1.7	0.0	
Unknown	9.1	10.3	6.8	8.3	

Table 3 demonstrated the results of frequency of numbers of eating behaviors according to the quartile of dietary knowledge score among college students. The fourth quartile with the highest dietary knowledge score showed a significantly highest frequency of adherence to 6 or more eating behaviors (*p* < 0.001). On the other hand, no significant relationship was found between knowledge of dietary habits and adherence to dietary diversity in the adolescent population (Table 4).

**Table 3:** Frequency of numbers of eating behaviors according to the quartile of dietary knowledge score.

College students	Quartile 1 (lowest)	Quartile 2	Quartile 3	Quartile 4 (highest)	*χ*^2^	*p*- value
Numbers of eating behaviors						
0	0.3	0.3	0.2	0.3	56.983	<0.001
1	2.0	1.7	1.5	1.7		
2	5.4	5.1	4.9	4.5		
3	14.3	11.9	10.6	11.0		
4	19.2	18.3	18.8	18.5		
5	22.9	24.6	22.8	21.9		
6	19.4	20.5	21.4	21.8		
7	11.8	12.8	15.0	13.8		
8	4.0	3.9	4.0	5.5		
9	0.7	0.8	0.8	1.0		

In this table demonstrated the results of frequency of numbers of eating behaviors according to the quartile of dietary knowledge score. The fourth quartile with the highest dietary knowledge score showed a significantly highest frequency of adherence to 6 or more eating behaviors (*p* < 0.001).

**Table 4:** Frequency of numbers of eating behaviors according to the quartile of dietary knowledge score.

Adolescent	Quartile 1 (lowest)	Quartile 2	Quartile 3	Quartile 4 (highest)	*χ*^2^	*p*- value
Numbers of eating behaviors						
0	2.1	4.4	2.8	5.6	12.872	0.612
1	14.0	14.7	10.2	9.7		
2	32.2	33.8	30.1	27.8		
3	26.6	17.6	18.8	22.2		
4	11.2	14.0	17.6	16.7		
5	14.0	15.4	20.5	18.1		

### 3.2 Multifactorial logistic regression analysis of dietary knowledge and likelihood of healthy eating behaviors among college students

In a sample of college students, this study examined the association between dietary knowledge and adherence to the number of different eating habits, with dietary knowledge as the independent variable and adherence to the number of different eating habits as the dependent variable. The results showed that there was positive association between dietary knowledge and adherence to 4–8 different eating habits.

The findings showed no significant association between dietary knowledge and adherence to two or more eating habits. In the crude model, the odds ratios (95% CI) for adherence to two or more eating habits were 1.14 (0.83, 1.57) for Q2, 1.35 (0.89, 2.04) for Q3, and 1.11 (0.77, 1.60) for Q4, compared to the reference group (Q1) (*p* = 0.426). After adjusting for confounders, the adjusted odds ratios (95% CI) were 1.09 (0.79, 1.50) for Q2, 1.24 (0.81, 1.88) for Q3, and 1.02 (0.70, 1.47) for Q4 (*p* = 0.783; Table 5).

**Table 5:** The associations between dietary knowledge and eating habits (≥2score).

College students	Number of participants	Number of healthy eating behaviors	Model 1^a^	Model 2^b^
Categories of dietary knowledge				
Quartile 1 (lowest)	3,040	2,971	1.000 (reference)	1.000 (reference)
Quartile 2	4,260	4,175	1.14 (0.83, 1.57)	1.09 (0.79, 1.50)
Quartile 3	2011	1977	1.35 (0.89, 2.04)	1.24 (0.81, 1.88)
Quartile 4 (highest)	2,545	2,493	1.11 (0.77, 1.60)	1.02 (0.70, 1.47)
*p*- for trend^d^	–	–	0.426	0.783

^a^Model 1: crude. ^b^Model 2: Adjusted for sex, age, annual family income, father and mother’s education (primary and below, junior high, high school, or junior college and above). ^c^Adjusted data are expressed as odds ratio (95% confidence intervals). ^d^*P* for trend were obtained using multivariate logistic regression analyses.

Similarly, no significant association was found between dietary knowledge and adherence to three or more eating habits. Crude odds ratios (95% CI) were 1.09 (0.91, 1.30), 1.18 (0.94, 1.47), and 1.20 (0.97, 1.47) for Q2, Q3, and Q4, respectively, compared to the reference group (Q1) (*p* = 0.066). After adjusting for confounding factors, the adjusted odds ratios (95% CI) were 1.05 (0.88, 1.26) for Q2, 1.09 (0.87, 1.36) for Q3, and 1.10 (0.89, 1.35) for Q4 (*p* = 0.345; Table 6).

**Table 6:** The associations between dietary knowledge and eating habits (≥3 score).

College students	Number of participants	Number of healthy eating behaviors	Model 1^a^	Model 2^b^
Categories of dietary knowledge				
Quartile 1 (lowest)	3,040	2,806	1.000 (reference)	1.000 (reference)
Quartile 2	4,260	3,957	1.09 (0.91, 1.30)	1.05 (0.88, 1.26)
Quartile 3	2011	1878	1.18 (0.94, 1.47)	1.09 (0.87, 1.36)
Quartile 4 (highest)	2,545	2,379	1.20 (0.97, 1.47)	1.10 (0.89, 1.35)
*p*- for trend^d^	–	–	0.066	0.345

^a^Model 1: crude. ^b^Model 2: Adjusted for sex, age, annual family income, father and mother’s education (primary and below, junior high, high school, or junior college and above). ^c^Adjusted data are expressed as odds ratio (95% confidence intervals). ^d^*P* for trend were obtained using multivariate logistic regression analyses.

However, a significant association emerged for adherence to four or more eating habits. The crude odds ratios (95% CI), compared to the reference group (Q1), were 1.20 (1.07, 1.35) for Q2, 1.35 (1.17, 1.56) for Q3, and 1.33 (1.16, 1.52) for Q4 (*p* < 0.001). After controlling for confounders, the adjusted odds ratios (95% CI) were 1.18 (1.05, 1.33), 1.28 (1.10, 1.48), and 1.24 (1.08, 1.42) for Q2, Q3, and Q4, respectively (*p* = 0.001; Table 7).

**Table 7:** The associations between dietary knowledge and eating habits (≥4 score).

College students	Number of participants	Number of healthy eating behaviors	Model 1^a^	Model 2^b^
Categories of dietary knowledge				
Quartile 1 (lowest)	3,040	2,372	1.000 (reference)	1.000 (reference)
Quartile 2	4,260	3,452	1.20 (1.07, 1.35)	1.18 (1.05, 1.33)
Quartile 3	2011	1,664	1.35 (1.17, 1.56)	1.28 (1.10, 1.48)
Quartile 4 (highest)	2,545	2099	1.33 (1.16, 1.52)	1.24 (1.08, 1.42)
*p*- for trend^d^	–	–	<0.001	0.001

^a^Model 1: crude. ^b^Model 2: Adjusted for sex, age, annual family income, father and mother’s education (primary and below, junior high, high school, or junior college and above). ^c^Adjusted data are expressed as odds ratio (95% confidence intervals). ^d^*P* for trend were obtained using multivariate logistic regression analyses.

For adherence to five or more eating habits, a positive association was also observed with dietary knowledge. The crude odds ratios (95% CI) for Q2, Q3, and Q4 were 1.18 (1.07, 1.30), 1.24 (1.11, 1.40), and 1.24 (1.12, 1.39), respectively, compared to the reference group (Q1) (*p* < 0.001). After adjusting for confounding factors, the adjusted odds ratios (95% CI) were 1.16 (1.06, 1.28) for Q2, 1.19 (1.06, 1.34) for Q3, and 1.18 (1.05, 1.31) for Q4 (*p* = 0.005; Table 8).

**Table 8:** The associations between dietary knowledge and eating habits (≥5 score).

College students	Number of participants	Number of healthy eating behaviors	Model 1^a^	Model 2^b^
Categories of dietary knowledge				
Quartile1 (lowest)	3,040	1787	1.000 (reference)	1.000 (reference)
Quartile2	4,260	2,671	1.18 (1.07, 1.30)	1.16 (1.06, 1.28)
Quartile3	2011	1,286	1.24 (1.11, 1.40)	1.19 (1.06, 1.34)
Quartile4 (highest)	2,545	1,627	1.24 (1.12, 1.39)	1.18 (1.05, 1.31)
*p*- for trend^d^	–	–	<0.001	0.005

^a^Model 1: crude. ^b^Model 2: Adjusted for sex, age, annual family income, father and mother’s education (primary and below, junior high, high school, or junior college and above). ^c^Adjusted data are expressed as odds ratio (95% confidence intervals). ^d^*P* for trend were obtained using multivariate logistic regression analyses.

The pattern held for adherence to six or more eating habits, where a significant positive association was noted. In comparison to the reference group (Q1), the crude odds ratios (95% CI) were 1.10 (1.00, 1.21) for Q2, 1.25 (1.11, 1.40) for Q3, and 1.29 (1.16, 1.44) for Q4 (*p* < 0.001). After controlling for confounders, the adjusted odds ratios (95% CI) were 1.09 (0.99, 1.20) for Q2, 1.19 (1.06, 1.34) for Q3, and 1.22 (1.09, 1.36) for Q4 (*p* < 0.001; Table 9).

**Table 9:** The associations between dietary knowledge and eating habits (≥6 score).

College students	Number of participants	Number of healthy eating behaviors	Model 1^a^	Model 2^b^
Categories of dietary knowledge				
Quartile 1 (lowest)	3,040	1,092	1.000 (reference)	1.000 (reference)
Quartile 2	4,260	1,621	1.10 (1.00, 1.21)	1.09 (0.99, 1.20)
Quartile 3	2011	828	1.25 (1.11, 1.40)	1.19 (1.06, 1.34)
Quartile 4 (highest)	2,545	1,069	1.29 (1.16, 1.44)	1.22 (1.09, 1.36)
*p*- for trend^d^	–	–	<0.001	<0.001

^a^Model 1: crude. ^b^Model 2: Adjusted for sex, age, annual family income, father and mother’s education (primary and below, junior high, high school, or junior college and above). ^c^Adjusted data are expressed as odds ratio (95% confidence intervals). ^d^*P* for trend were obtained using multivariate logistic regression analyses.

A similar significant association was observed for adherence to seven or more eating habits. The crude odds ratios (95% CI) for Q2, Q3, and Q4 were 1.07 (0.95, 1.21), 1.24 (1.07, 1.44), and 1.28 (1.12, 1.47), respectively, compared to Q1 (*p* < 0.001). After adjusting for confounding factors, the adjusted odds ratios (95% CI) were 1.06 (0.94, 1.21) for Q2, 1.18 (1.02, 1.38) for Q3, and 1.21 (1.05, 1.39) for Q4 (*p* = 0.003; Table 10).

**Table 10:** The associations between dietary knowledge and eating habits (≥7 score).

College students	Number of participants	Number of healthy eating behaviors	Model 1^a^	Model 2^b^
Categories of dietary knowledge				
Quartile 1 (lowest)	3,040	503	1.000 (reference)	1.000 (reference)
Quartile 2	4,260	746	1.07 (0.95, 1.21)	1.06 (0.94, 1.21)
Quartile 3	2011	397	1.24 (1.07, 1.44)	1.18 (1.02, 1.38)
Quartile 4 (highest)	2,545	515	1.28 (1.12, 1.47)	1.21 (1.05, 1.39)
*p*- for trend^d^	–	–	<0.001	0.003

^a^Model 1: crude. ^b^Model 2: Adjusted for sex, age, annual family income, father and mother’s education (primary and below, junior high, high school, or junior college and above). ^c^Adjusted data are expressed as odds ratio (95% confidence intervals). ^d^*P* for trend were obtained using multivariate logistic regression analyses.

A positive association between dietary knowledge and adherence to eight or more eating habits was also found. The crude odds ratios (95% CI), when compared to the reference group (Q1), were 1.00 (0.80, 1.25) for Q2, 1.01 (0.77, 1.31) for Q3, and 1.39 (1.11, 1.76) for Q4 (*p* = 0.004). After controlling for confounders, the adjusted odds ratios (95% CI) were 1.03 (0.82, 1.28) for Q2, 0.98 (0.75, 1.28) for Q3, and 1.32 (1.04, 1.66) for Q4 (*p* = 0.028; Table 11).

**Table 11:** The associations between dietary knowledge and eating habits (≥8 score).

College students	Number of participants	Number of healthy eating behaviors	Model 1^a^	Model 2^b^
Categories of dietary knowledge				
Quartile 1 (lowest)	3,040	144	1.000 (reference)	1.000 (reference)
Quartile 2	4,260	202	1.00 (0.80, 1.25)	1.03 (0.82, 1.28)
Quartile 3	2011	96	1.01 (0.77, 1.31)	0.98 (0.75, 1.28)
Quartile 4 (highest)	2,545	165	1.39 (1.11, 1.76)	1.32 (1.04, 1.66)
*p*- for trend^d^	–	–	0.004	0.028

^a^Model 1: crude. ^b^Model 2: Adjusted for sex, age, annual family income, father and mother’s education (primary and below, junior high, high school, or junior college and above). ^c^Adjusted data are expressed as odds ratio (95% confidence intervals). ^d^*P* for trend were obtained using multivariate logistic regression analyses.

In contrast, adherence to nine or more eating habits showed no significant association with dietary knowledge. The crude odds ratios (95% CI) were 1.17 (0.69, 1.99) for Q2, 1.10 (0.58, 2.10) for Q3, and 1.42 (0.80, 2.50) for Q4, compared to the reference group (Q1) (*p* = 0.276). After adjusting for confounders, the odds ratios (95% CI) were 1.18 (0.69, 2.02) for Q2, 1.01 (0.53, 1.95) for Q3, and 1.26 (0.71, 2.25) for Q4 (*p* = 0.560; Table 12).

**Table 12:** The associations between dietary knowledge and eating habits (≥9 score).

College students	Number of participants	Number of healthy eating behaviors	Model 1^a^	Model 2^b^
Categories of dietary knowledge				
Quartile 1 (lowest)	3,040	22	1.000 (reference)	1.000 (reference)
Quartile 2	4,260	36	1.17 (0.69, 1.99)	1.18 (0.69, 2.02)
Quartile 3	2011	16	1.10 (0.58, 2.10)	1.01 (0.53, 1.95)
Quartile 4 (highest)	2,545	26	1.42 (0.80, 2.50)	1.26 (0.71, 2.25)
*p*- for trend^d^	–	–	0.276	0.560

^a^Model 1: crude. ^b^Model 2: Adjusted for sex, age, annual family income, father and mother’s education (primary and below, junior high, high school, or junior college and above). ^c^Adjusted data are expressed as odds ratio (95% confidence intervals). ^d^*P* for trend were obtained using multivariate logistic regression analyses.

### 3.3 Multifactorial logistic regression analysis of dietary knowledge and likelihood of healthy eating behaviors among adolescents

In a sample of adolescent, this study examined the association between dietary knowledge and adherence to the number of different eating habits, with dietary knowledge as the independent variable and adherence to the number of different eating habits as the dependent variable.

Similarly, for two or more eating habits, no significant association with dietary knowledge was found. The crude odds ratios (95% CI) were 0.81 (0.44, 1.50), 1.28 (0.68, 2.38), and 1.06 (0.49, 2.32) for Q2, Q3, and Q4, respectively, compared to the reference group (Q1) (*p* = 0.491). After adjusting for confounders, the adjusted odds ratios (95% CI) were 0.85 (0.46, 1.60) for Q2, 1.16 (0.61, 2.20) for Q3, and 1.02 (0.46, 2.26) for Q4 (*p* = 0.705; Table 13).

**Table 13:** The associations between dietary knowledge and eating habits (≥2 score).

Adolescent	Number of participants	Number of healthy eating behaviors	Model 1^a^	Model 2^b^
Categories of dietary knowledge				
Quartile 1 (lowest)	143	120	1.000 (reference)	1.000 (reference)
Quartile 2	110	110	0.81 (0.44, 1.50)	0.85 (0.46, 1.60)
Quartile 3	153	153	1.28 (0.68, 2.38)	1.16 (0.61, 2.20)
Quartile 4 (highest)	61	61	1.06 (0.49, 2.32)	1.02 (0.46, 2.26)
*p*- for trend^d^	–	–	0.491	0.705

^a^Model 1: crude. ^b^Model 2: Adjusted for sex, age, annual family income, father and mother’s education (primary and below, junior high, high school, or junior college and above). ^c^Adjusted data are expressed as odds ratio (95% confidence intervals). ^d^*P* for trend were obtained using multivariate logistic regression analyses.

For adherence to three or more eating habits, the results showed no significant association between dietary knowledge and adherence. The crude odds ratios (95% CI), compared to the reference group (Q1) were 0.83 (0.52, 1.33) for Q2, 1.23 (0.79, 1.91) for Q3, and 1.23 (0.70, 2.18) for Q4 (*p* = 0.221). Adjusted odds ratios (95% CI) were 0.83 (0.52, 1.34) for Q2, 1.13 (0.72, 1.77) for Q3, and 1.19 (0.67, 2.11) for Q4 (*p* = 0.373; Table 14).

**Table 14:** The associations between dietary knowledge and eating habits (≥3 score).

Adolescent	Number of participants	Number of healthy eating behaviors	Model 1^a^	Model 2^b^
Categories of dietary knowledge				
Quartile 1 (lowest)	143	74	1.000 (reference)	1.000 (reference)
Quartile 2	110	64	0.83 (0.52, 1.33)	0.83 (0.52, 1.34)
Quartile 3	153	100	1.23 (0.79, 1.91)	1.13 (0.72, 1.77)
Quartile 4 (highest)	61	41	1.23 (0.70, 2.18)	1.19 (0.67, 2.11)
*p*- for trend^d^	–	–	0.221	0.373

^a^Model 1: crude. ^b^Model 2: Adjusted for sex, age, annual family income, father and mother’s education (primary and below, junior high, high school, or junior college and above). ^c^Adjusted data are expressed as odds ratio (95% confidence intervals). ^d^*P* for trend were obtained using multivariate logistic regression analyses.

Adherence to four or more eating habits did not show a significant association with dietary knowledge. The crude odds ratios (95% CI) were 1.24 (0.73, 2.10) for Q2, 1.83 (1.13, 2.97) for Q3, and 1.58 (0.86, 2.92) for Q4, compared with the reference group (Q1) (*p* = 0.025). Adjusted odds ratios (95% CI) after controlling for confounding factors were 1.27 (0.74, 2.18) for Q2, 1.68 (1.02, 2.77) for Q3, and 1.53 (0.82, 2.86) for Q4 (*p* = 0.059; Table 15).

**Table 15:** The associations between dietary knowledge and eating habits (≥4 score).

Adolescent	Number of participants	Number of healthy eating behaviors	Model 1^a^	Model 2^b^
Categories of dietary knowledge				
Quartile 1 (lowest)	143	36	1.000 (reference)	1.000 (reference)
Quartile 2	110	40	1.24 (0.73, 2.10)	1.27 (0.74, 2.18)
Quartile 3	153	67	1.83 (1.13, 2.97)	1.68 (1.02, 2.77)
Quartile 4 (highest)	61	25	1.58 (0.86, 2.92)	1.53 (0.82, 2.86)
*p*- for trend^d^	–	–	0.025	0.059

^a^Model 1: crude. ^b^Model 2: Adjusted for sex, age, annual family income, father and mother’s education (primary and below, junior high, high school, or junior college and above). ^c^Adjusted data are expressed as odds ratio (95% confidence intervals). ^d^*P* for trend were obtained using multivariate logistic regression analyses.

Finally, adherence to five or more eating habits revealed no significant association with dietary knowledge. The crude odds ratios (95% CI) for Q2, Q3, and Q4 were 1.12 (0.58, 2.18), 1.58 (0.87, 2.88), and 1.36 (0.63, 2.91), respectively, compared to Q1 (*p* = 0.182). After adjusting for confounders, the odds ratios (95% CI) were 1.19 (0.60, 2.33) for Q2, 1.53 (0.83, 2.83) for Q3, and 1.35 (0.62, 2.92) for Q4 (*p* = 0.238; Table 16).

**Table 16:** The associations between dietary knowledge and eating habits (≥5 score).

Adolescent	Number of participants	Number of healthy eating behaviors	Model 1^a^	Model 2^b^
Categories of dietary knowledge				
Quartile 1 (lowest)	143	20	1.000 (reference)	1.000 (reference)
Quartile 2	110	21	1.12 (0.58, 2.18)	1.19 (0.60, 2.33)
Quartile 3	153	36	1.58 (0.87, 2.88)	1.53 (0.83, 2.83)
Quartile 4 (highest)	61	13	1.36 (0.63, 2.91)	1.35 (0.62, 2.92)
*p*- for trend^d^	–	–	0.182	0.238

^a^Model 1: crude. ^b^Model 2: Adjusted for sex, age, annual family income, father and mother’s education (primary and below, junior high, high school, or junior college and above). ^c^Adjusted data are expressed as odds ratio (95% confidence intervals). ^d^*P* for trend were obtained using multivariate logistic regression analyses.

### 3.4 Multifactorial logistic regression analysis of dietary knowledge and likelihood of individual dietary habits among college students

This study explored the association between dietary knowledge and individual dietary habits among college students. The odds ratios (95% CI) for water consumption, comparing Q2, Q3, and Q4 to the reference group (Q1), were 0.98 (0.86, 1.12), 1.04 (0.89, 1.22), and 1.14 (0.98, 1.32), respectively (*p* = 0.055). After adjusting for confounding factors, the adjusted odds ratios (95% CI) for Q2, Q3, and Q4 versus Q1 were 1.02 (0.89, 1.17), 1.05 (0.90, 1.24), and 1.14 (0.98, 1.32), respectively (*p* = 0.084; Supplementary Table S1).

For egg consumption, the odds ratios (95% CI) for Q2, Q3, and Q4 compared to the reference group (Q1) were 1.01 (0.92, 1.11), 1.11 (0.99, 1.24), and 1.16 (1.04, 1.29), respectively (*p* = 0.002). After accounting for the confounders, the adjusted odds ratios (95% CI) were 1.00 (0.91, 1.10), 1.08 (0.96, 1.21), and 1.12 (1.01, 1.25), respectively (*p* = 0.016; Supplementary Table S2).

Regarding milk consumption, the odds ratios (95% CI) for Q2, Q3, and Q4 in comparison to Q1 were 1.04 (0.94, 1.16), 1.09 (0.96, 1.24), and 1.13 (1.01, 1.28), respectively (*p* = 0.031). After adjusting for confounding factors, the adjusted odds ratios (95% CI) were 1.04 (0.93, 1.15) for Q2, 1.06 (0.93, 1.21) for Q3, and 1.10 (0.98, 1.25) for Q4 (*p* = 0.110; Supplementary Table S3).

In terms of vegetable consumption, the odds ratios (95% CI) for Q2, Q3, and Q4 relative to Q1 were 1.32 (1.08, 1.61), 1.50 (1.16, 1.93), and 1.29 (1.03, 1.62), respectively (*p* = 0.015). Adjusted odds ratios (95% CI) for Q2, Q3, and Q4 were 1.33 (1.09, 1.62), 1.48 (1.15, 1.92), and 1.27 (1.01, 1.60), respectively (*p* = 0.028; Supplementary Table S4).

When examining fruit consumption, the odds ratios (95% CI) for Q2, Q3, and Q4 compared to Q1 were 1.20 (1.07, 1.35), 1.24 (1.08, 1.43), and 1.24 (1.09, 1.42), respectively (*p* = 0.001). After adjusting for confounders, the adjusted odds ratios (95% CI) were 1.16 (1.03, 1.31) for Q2, 1.18 (1.02, 1.37) for Q3, and 1.19 (1.04, 1.36) for Q4 (*p* = 0.012; Supplementary Table S5).

For red meat consumption, the odds ratios (95% CI) comparing Q2, Q3, and Q4 to Q1 were 1.19 (1.05, 1.34), 1.17 (1.01, 1.35), and 1.13 (0.99, 1.29), respectively (*p* = 0.113). After adjusting for confounders, the adjusted odds ratios (95% CI) were 1.16 (1.03, 1.31) for Q2, 1.10 (0.95, 1.28) for Q3, and 1.05 (0.92, 1.21) for Q4 (*p* = 0.639; Supplementary Table S6).

For soy and soy products, the odds ratios (95% CI) for Q2, Q3, and Q4 compared to the reference group (Q1) were 1.17 (1.05, 1.30), 1.25 (1.10, 1.42), and 1.16 (1.03, 1.30), respectively (*p* = 0.011). After adjusting for the confounding factors, the adjusted odds ratios (95% CI) were 1.15 (1.04, 1.28) for Q2, 1.20 (1.05, 1.37) for Q3, and 1.10 (0.97, 1.24) for Q4 (*p* = 0.112; Supplementary Table S7).

The analysis of seafood consumption revealed odds ratios (95% CI) of 0.97 (0.88, 1.06) for Q2, 1.04 (0.93, 1.17) for Q3, and 1.10 (0.99, 1.22) for Q4, compared to the reference group Q1 (*p* = 0.036). After controlling for confounders, the adjusted odds ratios (95% CI) were 0.96 (0.87, 1.06) for Q2, 1.00 (0.89, 1.12) for Q3, and 1.03 (0.92, 1.15) for Q4 (*p* = 0.459; Supplementary Table S8).

Lastly, for sugar-sweetened beverage consumption, the odds ratios (95% CI) for Q2, Q3, and Q4 compared to the reference group (Q1) were 1.05 (0.95, 1.16), 1.13 (1.00, 1.28), and 1.17 (1.04, 1.31), respectively (*p* = 0.004). After adjusting for confounding factors, the adjusted odds ratios (95% CI) were 1.05 (0.94, 1.16) for Q2, 1.12 (0.99, 1.27) for Q3, and 1.16 (1.03, 1.30) for Q4 (*p* = 0.007; Supplementary Table S9).

### 3.5 Multifactorial logistic regression analysis of dietary knowledge and likelihood of individual dietary habits among adolescent

This study investigated the association between dietary knowledge and individual dietary habits among adolescents. The odds ratios (95% CI) for vegetable consumption in Q2, Q3, and Q4, compared to the reference group (Q1), were 1.57 (0.98, 2.52), 2.78 (1.75, 4.42), and 2.01 (1.12, 3.59), respectively (*p* < 0.001). After adjusting for variables such as sex, age, annual family income, and parents’ education, the adjusted odds ratios (95% CI) for Q2, Q3, and Q4 compared to Q1 were 1.64 (1.01, 2.64), 2.62 (1.63, 4.20), and 1.97 (1.09, 3.55), respectively (*p* < 0.001; Supplementary Table S10).

For fruit consumption, the odds ratios (95% CI) for Q2, Q3, and Q4 relative to Q1 were 1.43 (0.83, 2.48), 1.82 (1.07, 3.09), and 2.41 (1.13, 5.16), respectively (*p* = 0.007). After adjusting for sex, age, annual family income, and parents’ education, the adjusted ratios (95% CI) were 1.51 (0.86, 2.63) for Q2, 1.89 (1.09, 3.27) for Q3, and 2.51 (1.16, 5.42) for Q4 (*p* = 0.006; Supplementary Table S11).

In terms of fast-food consumption, the odds ratios (95% CI) comparing Q2, Q3, and Q4 to the reference group (Q1) were 1.05 (0.65, 1.67), 1.24 (0.80, 1.93), and 1.38 (0.78, 2.45), respectively (*p* = 0.191). After adjusting for confounding factors such as sex, age, family income, and parents’ education, the adjusted odds ratios (95% CI) were 1.08 (0.67, 1.75) for Q2, 1.22 (0.77, 1.92) for Q3, and 1.38 (0.77, 2.47) for Q4 (*p* = 0.227; Supplementary Table S12).

For salty snack consumption, the odds ratios (95% CI) for Q2, Q3, and Q4 compared to Q1 were 0.74 (0.46, 1.18), 1.06 (0.68, 1.65), and 0.79 (0.45, 1.39), respectively (*p* = 0.863). After adjusting for sex, age, annual family income, and parents’ education, the adjusted odds ratios (95% CI) for were 0.73 (0.46, 1.17) for Q2, 0.99 (0.63, 1.55) for Q3, and 0.76 (0.43, 1.35) for Q4 (*p* = 0.659; Supplementary Table S13).

The analysis of soft drink and sugared fruit drink consumption revealed odds ratios (95% CI) of 0.52 (0.32, 0.84) for Q2, 0.55 (0.35, 0.86) for Q3, and 0.49 (0.27, 0.89) for Q4 compared to the reference group (*p* = 0.008). After adjusting for sex, age, annual family income, and parents’ education, the adjusted odds ratios (95% CI) were 0.51 (0.31, 0.84) for Q2, 0.48 (0.30, 0.77) for Q3, and 0.46 (0.25, 0.83) for Q4 (*p* = 0.002; Supplementary Table S14).

## 4 Discussion

This was the first study to examine the relationship between nutritional knowledge and dietary behavior based on the *Guidelines*. The results demonstrated that individuals with higher levels of nutritional knowledge tended to exhibit healthy eating patterns for individual food types and a greater propensity for diverse healthy dietary behaviors among college students. In contrast, similar association was not observed among adolescent. Thus, nutritional knowledge was associated with specific dietary behaviors and overall dietary diversity among college students.

The present findings were consistent with those of previous studies indicating that adults with good knowledge of the *Guidelines* exhibited significantly higher daily intake of grains, roots, vegetables, beans, tofu products, fruits, meats, poultry, eggs, and dairy products than those with poor knowledge (Feng Peng et al., 2019), and that primary and middle school students with good knowledge of the *Guidelines* exhibited better hydration habits and higher intake of dark green vegetables and eggs than those with poor knowledge (Huang FeiFei et al., 2015).

This relationship could be explained by the social cognitive theory, which suggests that individuals recognize patterns in their thinking, emotional responses, and behaviors and the conditions in which these occur. A previous study based on the social cognitive theory and health belief model found that individuals who understand how to change their behaviors and the modifiable aspects of their environment may implement corrective changes (Tougas et al., 2015). According to the social cognitive theory, individuals require a basic understanding of food and healthy eating conditions to effectively change dietary behavior. Individuals who obtain accurate dietary information and recognize the relationship between food intake and health adjust their dietary habits.

Previous study has found a positive correlation between health awareness and healthy dietary habits (Beydoun and Wang, 2008). Many studies have explored the association between dietary knowledge and quality and found that people recognized the usefulness of dietary guidelines, food composition, and expert advice for obtaining healthy dietary patterns (Bonaccio et al., 2013). These findings prompted strong endorsements of health education programs to inform dietary choices. Therefore, a lack of knowledge could lead to unhealthy eating habits, which is supported by sociological theories, such as the social cognitive theory. A longitudinal follow-up survey in China revealed that awareness of the Guidelines among adults increased from 7.8% in 2004 to 24.4% in 2011, indicating that more than 70% of Chinese adults were unaware of the Guidelines (Huang FeiFei et al., 2015). This lack of awareness may be related to unhealthy eating habits. Surprisingly, similar association was not observed among adolescent. It can be reasonably assumed that guardians or school cafeteria staff exert a significant influence on adolescents’ dietary habits, given the typical provision of food to this age group by their respective guardians or the school. As a consequence, adolescents are unable to select a range of food items in accordance with their personal preferences.

In China, dietary guidelines significantly enhanced public understanding of food and strengthened self-awareness of the necessity of a balanced diet. The significance of nutritional knowledge was particularly evident when the results were stratified by education level (Supplementary Table S13). It is reasonable to believe that individuals with a higher degree of education may be more inclined to be aware of the nutritional quality of food and thus to choose healthy diets.

### 4.1 Limitations

This study had several limitations. First, the limited food preference questionnaire hindered our ability to assess the multidimensional nature of dietary preferences. Future studies should explore dietary knowledge based on the *Guidelines* and multidimensional food preferences. Second, due to the cross-sectional design of this study, we could not determine the causal association between dietary knowledge and behaviors. Future cohort studies should examine the association between dietary knowledge and eating behaviors. Third, this study employed a questionnaire on dietary knowledge and eating behaviors that has been extensively used in previous research (Ma et al., 2024; Liu et al., 2023). However, the use of self-report data on congenital deficiencies in the questionnaire limits the accuracy of the investigation into the dietary knowledge and eating behaviors of the participants. Fourth, the adoption of healthy eating behaviors or the acquisition of knowledge about healthy diets over time can result in an excessive preoccupation with healthy eating (Douma et al., 2021), which in turn may give rise to the development of specific healthy eating habits. Such habits may contribute to the occurrence of eating disorders when they become a dominant focus (McCartney, 2016). It would be beneficial for future longitudinal follow-up studies to focus on whether long-term adherence to healthy eating habits, or an unhealthy obsession with healthy eating habits, leads to the development of eating disorders.

## 5 Conclusion

This study revealed a significant association between dietary knowledge based on the *Guidelines* and adherence to healthy dietary behaviors among college students in China. That is to say, the higher the level of dietary knowledge based on the Guidelines among college students, the healthier the dietary behaviors they tend to adopt in their daily lives. These findings indicate the necessity of developing educational interventions based on the Guidelines to enhance dietary knowledge among individuals with limited dietary knowledge. Such interventions could facilitate the acquisition of essential health-related knowledge and strengthen motivation to engage in healthy dietary behaviors. Future studies should employ longitudinal prospective designs or randomized controlled trials in order to establish a causal association between dietary knowledge based on the Guidelines and healthy dietary behaviors.

## Data Availability

The raw data supporting the conclusions of this article will be made available by the authors, without undue reservation.
