# Supplementary material for: The association between dietary knowledge based on the Chinese Dietary Guidelines and adherence to healthy dietary habits: a large-scale cross-sectional study
Source: Front Psychol. 2024 Oct 18;15:1453815. doi: 10.3389/fpsyg.2024.1453815 (PMC11528709; doi:10.3389/fpsyg.2024.1453815)
Supplement: Supplementary file 1 [file Data_Sheet_1.docx]

Supplementary Material

**Supplementary Table 1 The associations between dietary knowledge and water intake (≥1500 ml per day)**

| **College students** | **Number of participants** | **Number of healthy eating behaviors** | **Model 1 ^a^** | **Model 2 ^b^** |
| --- | --- | --- | --- | --- |
| Categories of dietary knowledge | |  |  |  |
| Quartile1 (lowest) | 3040 | 441 | 1.000 (reference) | 1.000 (reference) |
| Quartile2 | 4260 | 608 | 0.98 (0.86, 1.12) | 1.02 (0.89, 1.17) |
| Quartile3 | 2011 | 301 | 1.04 (0.89, 1.22) | 1.05 (0.90, 1.24) |
| Quartile4 (highest) | 2545 | 412 | 1.14 (0.98, 1.32) | 1.14 (0.98, 1.32) |
| P for trend ^d^ | — | — | 0.055 | 0.084 |

^a^ Model 1: crude

^b^ Model 2: Adjusted for sex, age, annual family income, father and mother’s education (primary and below, junior high, high school, or junior college and above).

^c^ Adjusted data are expressed as odds ratio (95% confidence intervals).

^d^ P for trend were obtained using multivariate logistic regression analyses

**Supplementary Table 2 The associations between dietary knowledge and** **egg intake (at least one per day)**

| **College students** | **Number of participants** | **Number of healthy eating behaviors** | **Model 1 ^a^** | **Model 2 ^b^** |
| --- | --- | --- | --- | --- |
| Categories of dietary knowledge | |  |  |  |
| Quartile1 (lowest) | 3040 | 1659 | 1.000 (reference) | 1.000 (reference) |
| Quartile2 | 4260 | 2333 | 1.01 (0.92, 1.11) | 1.00 (0.91, 1.10) |
| Quartile3 | 2011 | 1148 | 1.11 (0.99, 1.24) | 1.08 (0.96, 1.21) |
| Quartile4 (highest) | 2545 | 1482 | 1.16 (1.04, 1.29) | 1.12 (1.01, 1.25) |
| P for trend ^d^ | — | — | 0.002 | 0.016 |

^a^ Model 1: crude

^b^ Model 2: Adjusted for sex, age, annual family income, father and mother’s education (primary and below, junior high, high school, or junior college and above).

^c^ Adjusted data are expressed as odds ratio (95% confidence intervals).

^d^ P for trend were obtained using multivariate logistic regression analyses

**Supplementary Table 3 The associations between dietary knowledge and milk intake (at least 250 ml per day)**

| **College students** | **Number of participants** | **Number of healthy eating behaviors** | **Model 1 ^a^** | **Model 2 ^b^** |
| --- | --- | --- | --- | --- |
| Categories of dietary knowledge | |  |  |  |
| Quartile1 (lowest) | 3040 | 749 | 1.000 (reference) | 1.000 (reference) |
| Quartile2 | 4260 | 1083 | 1.04 (0.94, 1.16) | 1.04 (0.93, 1.15) |
| Quartile3 | 2011 | 528 | 1.09 (0.96, 1.24) | 1.06 (0.93, 1.21) |
| Quartile4 (highest) | 2545 | 688 | 1.13 (1.01, 1.28) | 1.10 (0.98, 1.25) |
| P for trend ^d^ | — | — | 0.031 | 0.110 |

^a^ Model 1: crude

^b^ Model 2: Adjusted for sex, age, annual family income, father and mother’s education (primary and below, junior high, high school, or junior college and above).

^c^ Adjusted data are expressed as odds ratio (95% confidence intervals).

^d^ P for trend were obtained using multivariate logistic regression analyses

**Supplementary Table 4 The associations between dietary knowledge and vegetable intake (at least once per day)**

| **College students** | **Number of participants** | **Number of healthy eating behaviors** | **Model 1 ^a^** | **Model 2 ^b^** |
| --- | --- | --- | --- | --- |
| Categories of dietary knowledge | |  |  |  |
| Quartile1 (lowest) | 3040 | 2841 | 1.000 (reference) | 1.000 (reference) |
| Quartile2 | 4260 | 4045 | 1.32 (1.08, 1.61) | 1.33 (1.09, 1.62) |
| Quartile3 | 2011 | 1921 | 1.50 (1.16, 1.93) | 1.48 (1.15, 1.92) |
| Quartile4 (highest) | 2545 | 2414 | 1.29 (1.03, 1.62) | 1.27 (1.01, 1.60) |
| P for trend ^d^ | — | — | 0.015 | 0.028 |

^a^ Model 1: crude

^b^ Model 2: Adjusted for sex, age, annual family income, father and mother’s education (primary and below, junior high, high school, or junior college and above).

^c^ Adjusted data are expressed as odds ratio (95% confidence intervals).

^d^ P for trend were obtained using multivariate logistic regression analyses

**Supplementary Table 5 The associations between dietary knowledge and fruits intake (at least once per day)**

| **College students** | **Number of participants** | **Number of healthy eating behaviors** | **Model 1 ^a^** | **Model 2 ^b^** |
| --- | --- | --- | --- | --- |
| Categories of dietary knowledge | |  |  |  |
| Quartile1 (lowest) | 3040 | 2376 | 1.000 (reference) | 1.000 (reference) |
| Quartile2 | 4260 | 3455 | 1.20 (1.07, 1.35) | 1.16 (1.03, 1.31) |
| Quartile3 | 2011 | 1641 | 1.24 (1.08, 1.43) | 1.18 (1.02, 1.37) |
| Quartile4 (highest) | 2545 | 2078 | 1.24 (1.09, 1.42) | 1.19 (1.04, 1.36) |
| P for trend ^d^ | — | — | 0.001 | 0.012 |

^a^ Model 1: crude

^b^ Model 2: Adjusted for sex, age, annual family income, father and mother’s education (primary and below, junior high, high school, or junior college and above).

^c^ Adjusted data are expressed as odds ratio (95% confidence intervals).

^d^ P for trend were obtained using multivariate logistic regression analyses

**Supplementary Table 6 The associations between dietary knowledge and red meat intake (at least twice per week)**

| **College students** | **Number of participants** | **Number of healthy eating behaviors** | **Model 1 ^a^** | **Model 2 ^b^** |
| --- | --- | --- | --- | --- |
| Categories of dietary knowledge | |  |  |  |
| Quartile1 (lowest) | 3040 | 2425 | 1.000 (reference) | 1.000 (reference) |
| Quartile2 | 4260 | 3510 | 1.19 (1.05, 1.34) | 1.16 (1.03, 1.31) |
| Quartile3 | 2011 | 1652 | 1.17 (1.01, 1.35) | 1.10 (0.95, 1.28) |
| Quartile4 (highest) | 2545 | 2078 | 1.13 (0.99, 1.29) | 1.05 (0.92, 1.21) |
| P for trend ^d^ | — | — | 0.113 | 0.639 |

^a^ Model 1: crude

^b^ Model 2: Adjusted for sex, age, annual family income, father and mother’s education (primary and below, junior high, high school, or junior college and above).

^c^ Adjusted data are expressed as odds ratio (95% confidence intervals).

^d^ P for trend were obtained using multivariate logistic regression analyses

**Supplementary Table 7 The associations between dietary knowledge and soy and soy products intake (at least twice per week)**

| **College students** | **Number of participants** | **Number of healthy eating behaviors** | **Model 1 ^a^** | **Model 2 ^b^** |
| --- | --- | --- | --- | --- |
| Categories of dietary knowledge | |  |  |  |
| Quartile1 (lowest) | 3040 | 2195 | 1.000 (reference) | 1.000 (reference) |
| Quartile2 | 4260 | 3204 | 1.17 (1.05, 1.30) | 1.15 (1.04, 1.28) |
| Quartile3 | 2011 | 1537 | 1.25 (1.10, 1.42) | 1.20 (1.05, 1.37) |
| Quartile4 (highest) | 2545 | 1909 | 1.16 (1.03, 1.30) | 1.10 (0.97, 1.24) |
| P for trend ^d^ | — | — | 0.011 | 0.112 |

^a^ Model 1: crude

^b^ Model 2: Adjusted for sex, age, annual family income, father and mother’s education (primary and below, junior high, high school, or junior college and above).

^c^ Adjusted data are expressed as odds ratio (95% confidence intervals).

^d^ P for trend were obtained using multivariate logistic regression analyses

**Supplementary Table 8 The associations between dietary knowledge and seafood intake (at least twice per week)**

| **College students** | **Number of participants** | **Number of healthy eating behaviors** | **Model 1 ^a^** | **Model 2 ^b^** |
| --- | --- | --- | --- | --- |
| Categories of dietary knowledge | |  |  |  |
| Quartile1 (lowest) | 3040 | 1195 | 1.000 (reference) | 1.000 (reference) |
| Quartile2 | 4260 | 1641 | 0.97 (0.88, 1.06) | 0.96 (0.87, 1.06) |
| Quartile3 | 2011 | 811 | 1.04 (0.93, 1.17) | 1.00 (0.89, 1.12) |
| Quartile4 (highest) | 2545 | 1058 | 1.10 (0.99, 1.22) | 1.03 (0.92, 1.15) |
| P for trend ^d^ | — | — | 0.036 | 0.459 |

^a^ Model 1: crude

^b^ Model 2: Adjusted for sex, age, annual family income, father and mother’s education (primary and below, junior high, high school, or junior college and above).

^c^ Adjusted data are expressed as odds ratio (95% confidence intervals).

^d^ P for trend were obtained using multivariate logistic regression analyses

**Supplementary Table 9 The associations between dietary knowledge and sugar-sweetened beverages intake (never consume)**

| **College students** | **Number of participants** | **Number of healthy eating behaviors** | **Model 1 ^a^** | **Model 2 ^b^** |
| --- | --- | --- | --- | --- |
| Categories of dietary knowledge | |  |  |  |
| Quartile1 (lowest) | 3040 | 847 | 1.000 (reference) | 1.000 (reference) |
| Quartile2 | 4260 | 1229 | 1.05 (0.95, 1.16) | 1.05 (0.94, 1.16) |
| Quartile3 | 2011 | 610 | 1.13 (1.00, 1.28) | 1.12 (0.99, 1.27) |
| Quartile4 (highest) | 2545 | 791 | 1.17 (1.04, 1.31) | 1.16 (1.03, 1.30) |
| P for trend ^d^ | — | — | 0.004 | 0.007 |

^a^ Model 1: crude

^b^ Model 2: Adjusted for sex, age, annual family income, father and mother’s education (primary and below, junior high, high school, or junior college and above).

^c^ Adjusted data are expressed as odds ratio (95% confidence intervals).

^d^ P for trend were obtained using multivariate logistic regression analyses

**Supplementary Table 10 The associations between dietary knowledge and vegetables intake**

| **Adolescent** | **Number of participants** | **Number of healthy eating behaviors** | **Model 1 ^a^** | **Model 2 ^b^** |
| --- | --- | --- | --- | --- |
| Categories of dietary knowledge | |  |  |  |
| Quartile1 (lowest) | 143 | 67 | 1.000 (reference) | 1.000 (reference) |
| Quartile2 | 110 | 79 | 1.57 (0.98, 2.52) | 1.64 (1.01, 2.64) |
| Quartile3 | 153 | 125 | 2.78 (1.75, 4.42) | 2.62 (1.63, 4.20) |
| Quartile4 (highest) | 61 | 46 | 2.01 (1.12, 3.59) | 1.97 (1.09, 3.55) |
| P for trend ^d^ | — | — | <0.001 | <0.001 |

^a^ Model 1: crude

^b^ Model 2: Adjusted for sex, age, annual family income, father and mother’s education (primary and below, junior high, high school, or junior college and above).

^c^ Adjusted data are expressed as odds ratio (95% confidence intervals).

^d^ P for trend were obtained using multivariate logistic regression analyses

**Supplementary Table 11 The associations between dietary knowledge and fruits intake**

| **Adolescent** | **Number of participants** | **Number of healthy eating behaviors** | **Model 1 ^a^** | **Model 2 ^b^** |
| --- | --- | --- | --- | --- |
| Categories of dietary knowledge | |  |  |  |
| Quartile1 (lowest) | 143 | 103 | 1.000 (reference) | 1.000 (reference) |
| Quartile2 | 110 | 107 | 1.43 (0.83, 2.48) | 1.51 (0.86, 2.63) |
| Quartile3 | 153 | 145 | 1.82 (1.07, 3.09) | 1.89 (1.09, 3.27) |
| Quartile4 (highest) | 61 | 62 | 2.41 (1.13, 5.16) | 2.51 (1.16, 5.42) |
| P for trend ^d^ | — | — | 0.007 | 0.006 |

^a^ Model 1: crude

^b^ Model 2: Adjusted for sex, age, annual family income, father and mother’s education (primary and below, junior high, high school, or junior college and above).

^c^ Adjusted data are expressed as odds ratio (95% confidence intervals).

^d^ P for trend were obtained using multivariate logistic regression analyses

**Supplementary Table 12 The associations between dietary knowledge and fast-food intake**

| **Adolescent** | **Number of participants** | **Number of healthy eating behaviors** | **Model 1 ^a^** | **Model 2 ^b^** |
| --- | --- | --- | --- | --- |
| Categories of dietary knowledge | |  |  |  |
| Quartile1 (lowest) | 143 | 72 | 1.000 (reference) | 1.000 (reference) |
| Quartile2 | 110 | 70 | 1.05 (0.65, 1.67) | 1.08 (0.67, 1.75) |
| Quartile3 | 153 | 98 | 1.24 (0.80, 1.93) | 1.22 (0.77, 1.92) |
| Quartile4 (highest) | 61 | 42 | 1.38 (0.78, 2.45) | 1.38 (0.77, 2.47) |
| P for trend ^d^ | — | — | 0.191 | 0.227 |

^a^ Model 1: crude

^b^ Model 2: Adjusted for sex, age, annual family income, father and mother’s education (primary and below, junior high, high school, or junior college and above).

^c^ Adjusted data are expressed as odds ratio (95% confidence intervals).

^d^ P for trend were obtained using multivariate logistic regression analyses

**Supplementary Table 13 The associations between dietary knowledge and salty snack food intake**

| **Adolescent** | **Number of participants** | **Number of healthy eating behaviors** | **Model 1 ^a^** | **Model 2 ^b^** |
| --- | --- | --- | --- | --- |
| Categories of dietary knowledge | |  |  |  |
| Quartile1 (lowest) | 143 | 76 | 1.000 (reference) | 1.000 (reference) |
| Quartile2 | 110 | 62 | 0.74 (0.46, 1.18) | 0.73 (0.46, 1.17) |
| Quartile3 | 153 | 96 | 1.06 (0.68, 1.65) | 0.99 (0.63, 1.55) |
| Quartile4 (highest) | 61 | 34 | 0.79 (0.45, 1.39) | 0.76 (0.43, 1.35) |
| P for trend ^d^ | — | — | 0.863 | 0.659 |

^a^ Model 1: crude

^b^ Model 2: Adjusted for sex, age, annual family income, father and mother’s education (primary and below, junior high, high school, or junior college and above).

^c^ Adjusted data are expressed as odds ratio (95% confidence intervals).

^d^ P for trend were obtained using multivariate logistic regression analyses

**Supplementary Table 14 The associations between dietary knowledge and soft drinks and sugared fruit drinks intake**

| **Adolescent** | **Number of participants** | **Number of healthy eating behaviors** | **Model 1 ^a^** | **Model 2 ^b^** |
| --- | --- | --- | --- | --- |
| Categories of dietary knowledge | |  |  |  |
| Quartile1 (lowest) | 143 | 72 | 1.000 (reference) | 1.000 (reference) |
| Quartile2 | 110 | 47 | 0.52 (0.32, 0.84) | 0.51 (0.31, 0.84) |
| Quartile3 | 153 | 63 | 0.55 (0.35, 0.86) | 0.48 (0.30, 0.77) |
| Quartile4 (highest) | 61 | 24 | 0.49 (0.27, 0.89) | 0.46 (0.25, 0.83) |
| P for trend ^d^ | — | — | 0.008 | 0.002 |

^a^ Model 1: crude

^b^ Model 2: Adjusted for sex, age, annual family income, father and mother’s education (primary and below, junior high, high school, or junior college and above).

^c^ Adjusted data are expressed as odds ratio (95% confidence intervals).

^d^ P for trend were obtained using multivariate logistic regression analyses.
